# Monitoring cotton root rot by synthetic Sentinel-2 NDVI time series using improved spatial and temporal data fusion

**DOI:** 10.1038/s41598-018-20156-z

**Published:** 2018-01-31

**Authors:** Mingquan Wu, Chenghai Yang, Xiaoyu Song, Wesley Clint Hoffmann, Wenjiang Huang, Zheng Niu, Changyao Wang, Wang Li, Bo Yu

**Affiliations:** 1 The State Key Laboratory of Remote Sensing Science, Institute of Remote Sensing and Digital Earth, Chinese Academy of Sciences, P.O. Box 9718, 20 Datun Road, Chaoyang, Beijing, 100101 China; 2 USDA-Agricultural Research Service, Aerial Application Technology Research Unit, 3103 F & B Road, College Station, TX 77845 USA; 3 Beijing Research Center for Information Technology in Agriculture, Beijing Academy of Agriculture and Forestry Sciences, Beijing, 100097 China; 4 Laboratory of Digital Earth Sciences, Institute of Remote Sensing and Digital Earth, Chinese Academy of Sciences, Beijing, 100094 China

## Abstract

To better understand the progression of cotton root rot within the season, time series monitoring is required. In this study, an improved spatial and temporal data fusion approach (ISTDFA) was employed to combine 250-m Moderate Resolution Imaging Spectroradiometer (MODIS) Normalized Different Vegetation Index (NDVI) and 10-m Sentinetl-2 NDVI data to generate a synthetic Sentinel-2 NDVI time series for monitoring this disease. Then, the phenology of healthy cotton and infected cotton was modeled using a logistic model. Finally, several phenology parameters, including the onset day of greenness minimum (OGM), growing season length (GLS), onset of greenness increase (OGI), max NDVI value, and integral area of the phenology curve, were calculated. The results showed that ISTDFA could be used to combine time series MODIS and Sentinel-2 NDVI data with a correlation coefficient of 0.893. The logistic model could describe the phenology curves with R-squared values from 0.791 to 0.969. Moreover, the phenology curve of infected cotton showed a significant difference from that of healthy cotton. The max NDVI value, OGM, GSL and the integral area of the phenology curve for infected cotton were reduced by 0.045, 30 days, 22 days, and 18.54%, respectively, compared with those for healthy cotton.

## Introduction

Cotton root rot, also known as Phymatotrichum root rot or Texas root rot, is a serious cotton disease that causes sudden wilt and death of affected cotton, and results in a significant reduction of yield^1^. A commercial formulation of a new fungicide, flutriafol (FMC Corporation, Philadelphia, PA), which was evaluated by both field evaluation and airborne imaging technology, was found to control cotton root rot effectively^2,3^. However, flutriafol is expensive. Remote sensing, in particular airborne remote sensing, has been proved effective for mapping the disease so that variable rate technology can be used to treat the disease more economically^4^. The infected areas of cotton root rot in fields are generally stable and do not change significantly over time, so the fungicide can be applied only in these infected areas^4^.

Cotton root rot causes a spectral change in cotton that makes it suitable for mapping by remote sensing^2,5^. Airborne imagery has been proved effective in the identification of the infected areas of cotton root rot^1^. Yang *et al*. evaluated different spectral measures derived from airborne multispectral imagery for detecting cotton root rot, compared airborne multispectral and hyperspectral imagery for mapping this disease, and assessed unsupervised and supervised image classification methods for quantifying root rot-infected areas^1,6,7^. Zhang *et al*. designed a near real-time high-resolution airborne camera for mapping cotton root rot^8^. Song *et al*. combined fuzzy set theory and nonlinear stretching enhancement for unsupervised classification of cotton root rot^9^. By identifying the infected areas with airborne imagery, cotton root rot can be treated only in the infected area, thus reducing fungicide costs for the farmer.

In previous studies, airborne imagery has been mainly used to map the extent of cotton root rot infections near the end of the growing season when cotton root rot is fully pronounced for the season. Yang *et al*. also used airborne imagery for monitoring the progression of the infections within cotton fields during a growing season^4^. However, no satellite imagery has been evaluated for time series monitoring of cotton root rot.

To obtain time series high/medium spatial resolution remote sensing data, two approaches have been used in the remote sensing community. First, new high/medium spatial resolution satellites with significantly shorter revisit times launched in recent years have been used. For example, the RapidEye satellite sensor can acquire 5-m images every day, the Gaofen-1 (GF-1) wide field-of-view camera (WFV) provides 16-m images every two days, and the Sentinel-2 constellation provides 10-m images every five days after Sentinel-2B is launched on March 7, 2017. The short return cycle of these sensors makes them highly suitable for time series monitoring^10,11^. Second, several spatial and temporal data fusion methods have been proposed to combine high/medium spatial resolution remote sensing data with high temporal resolution data to generate time series high/medium spatial resolution data. Gao *et al*. proposed the spatial and temporal adaptive reflectance fusion model (STARFM) to blend Moderate Resolution Imaging Spectroradiometer (MODIS) and Landsat surface reflectance^12^. Zhang *et al*. proposed an enhanced spatial and temporal adaptive reflectance fusion model (ESTARFM) for complex areas^13^. Spatial and temporal fusion methods based on unmixing theory were also proposed. Huang *et al*. proposed a spatio-temporal reflectance fusion method to blend MODIS and Landsat Enhanced Thematic Mapper Plus (ETM+) imagery^14^. Wu *et al*. proposed a spatial and temporal data fusion approach (STDFA) and improved it to address the problem of spatial variability of surface reflectance^15,16^. Xie *et al*. improved STARFM using an unmixing-based method^17^. Liao *et al*. proposed a Normalized Different Vegetation Index (NDVI)-Bayesian spatiotemporal fusion model (NDVI-BSFM) to build frequent Landsat-like NDVI datasets by integrating MODIS and Landsat NDVI^18^. Zhang *et al*. compared several methods including STARFM, ESTARFM and non-linear methods, and found that non-linear methods like the sparse representation-based spatio-temporal reflectance fusion model are more suitable to monitor land-cover changes in addition to phonological changes^19^. To improve the spatial resolution of medium spatial resolution land surface phenology, several studies combined MODIS with Landsat reflectance^20–22^ and Wu *et al*. combined GF-1 WFV, Landsat, and MODIS data^11^. Wu *et al*. demonstrated that synthetic Landsat NDVI time series generated by improved STDFA (ISTDFA) can monitor the phenology of vegetation at a spatial resolution of 30-m successfully^23^. For land surface dynamics monitoring, Hilker *et al*. proposed a spatial temporal adaptive algorithm for mapping reflectance change (STAARCH) based on STARFM^24^, Zhu *et al*. proposed a flexible spatiotemporal data fusion (FSDAF) method^25^, and Lu *et al*. proposed an object-based data blending model^26^.

The spatial and temporal data fusion technology provides a method to generate time series high spatial resolution data for time series cotton root rot monitoring. However, most of these methods are focused on fusion MODIS data with medium resolution data (Landsat and GF-1 WFV), which are too coarse for cotton root rot monitoring. Thus, there is a need to combine higher spatial resolution data with MODIS for cotton root rot monitoring. Moreover, the Sentinal-2 data is a good selection which has a very similar visible and near-infrared (NIR) bands with MODIS and Landsat and a high spatial resolution (10-m) for these bands. For the time series monitoring of cotton root rot, the objectives of this study were to^1^ test the ability of an improved spatial and temporal data fusion approach (ISTDFM) in the fusion of MODIS and Sentinal-2 NDVI data; and^2^ monitor cotton root rot progression with the synthetic Sentinal-2 NDVI time series generated by ISTDFM.

## Results

### Fusion results of ISDFA

**Figure 1:**
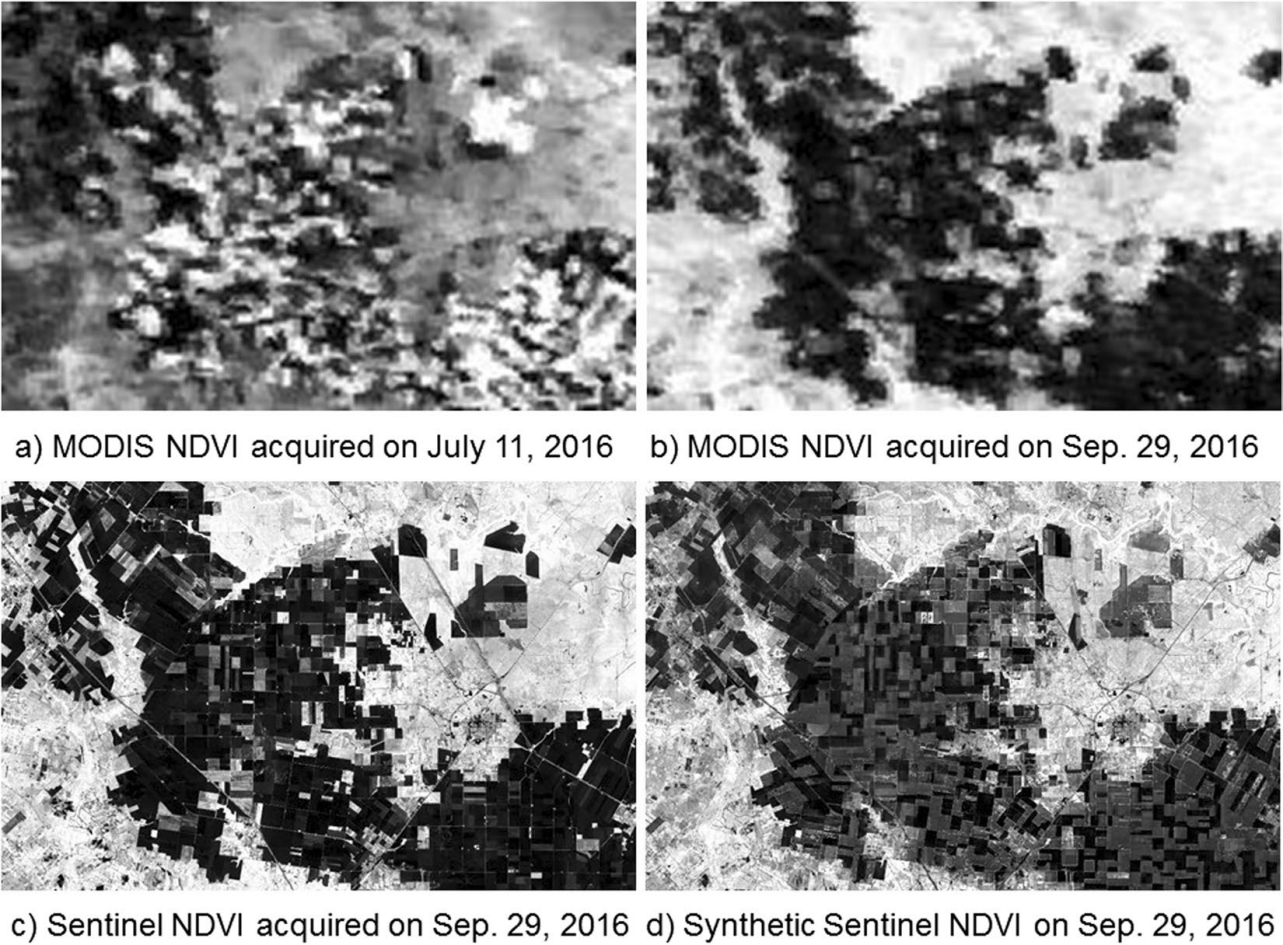
Fusion results of ISTDFA. (Figure (**a**–**d**) were calculated from MODIS images and Sentinal-2 imagery which were downloaded from the web page of the U.S. Geological Survey (USGS) Global Visualization Viewer (http://glovis.usgs.gov/); Figure (**a**–**d**) were combined to one Figure using Powerpoint 2013 (Microsoft Inc., Redmond, WA, USA, https://www.microsoft.com)).

Sixty synthetic 10-m Sentinel-2 NDVI images from January 1 to September 30, 2016, were generated by ISTDFA. Actual 10-m Sentinal-2 NDVI acquired on September 29, 2016 was used to evaluate the accuracy of ISTDFA. Figure 1a,b show the MODIS NDVI images on July 11 and September 30, 2016, respectively. Figure 1c shows the actual sentinel-2 NDVI image acquired on September 29, 2016. Figure 1d shows synthetic Sentinel-2 NDVI image on September 29, 2016. By comparing Fig. 1a,c, it can be found that the NDVI of crop land changed significantly. This is because cotton was harvested and the land cover types changed to bare land. By comparing Fig. 1c,d, it can be found that the synthetic and actual Sentinel-2 NDVI images are similar in most of the land cover types. However, the NDVI images of the areas where the land cover types changed from cotton to bare land show some differences. The NDVI values of these areas in the synthetic Sentinel-2 NDVI image were higher than those in the actual Sentinel-2 NDVI image. Reasons for this were discussed in the phenology monitoring of cotton root rot section. Although some differences were found between the synthetic and actual Sentinel-2 NDVIs, the changes (reflectance of cotton to reflectance of bare land) could still be detected by ISTDFA (Fig. 1a,d).

**Figure 2:**
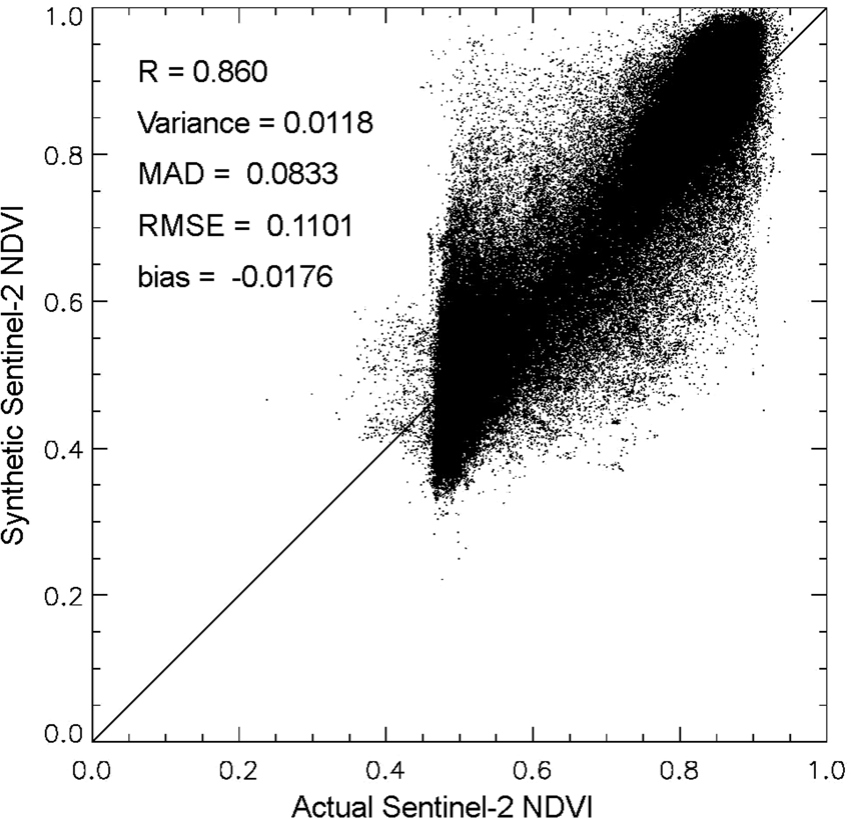
Scatter plot between actual and synthetic Sentinel-2 NDVI on September 29, 2016.

Figure 2 shows the scatter plot between the actual Sentinel-2 NDVI and synthetic Sentinel-2 NDVI along with the accuracy evaluation parameters (correlation coefficient (*R*), variance, mean absolute difference (*MAD*), *bias*, and root mean squared error (*RMSE)*). It can be seen that there is a high similarity between the actual Sentinel-2 NDVI and the synthetic Sentinel-2 NDVI with an *R*-value of 0.860. Most of the pixels are located near the 1:1 line in the scatter plot.

### Results of time series monitoring of cotton root rot

#### Calibration results of synthetic Sentinel-2 NDVI time series

**Figure 3:**
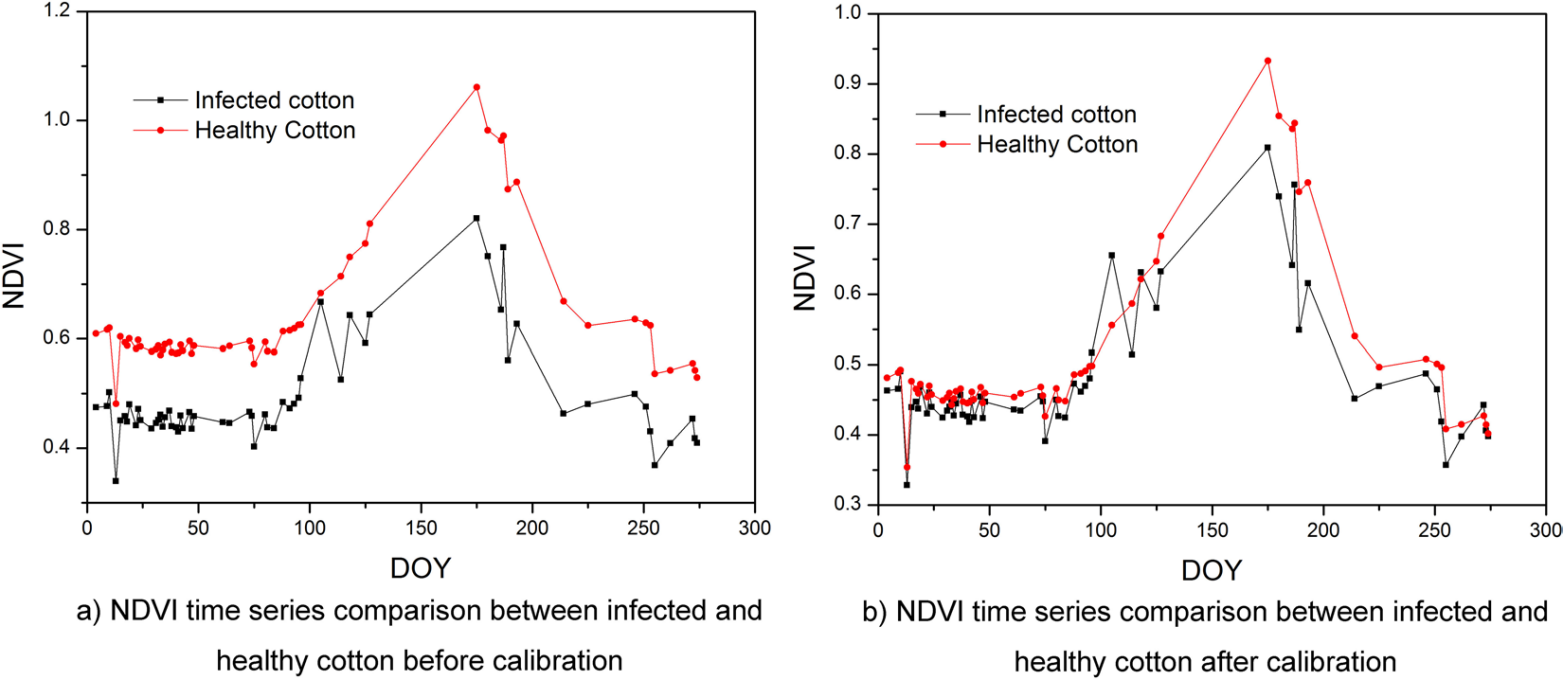
NDVI time series comparison between infected and healthy cotton (**a**) before calibration and (**b**) after calibration.

Figure 3 shows the NDVI time series comparison between infected and healthy cotton before calibration and after calibration. Figure 3a shows that the NDVI of healthy cotton was higher than that of infected cotton systematically before calibration at the early part of the year when cotton had not been sown and the cotton fields were still bare, due to the reasons explained in method section. Before calibration, the mean NDVI of healthy cotton from January 1 to March 14 was 0.585 while it was 0.451 for infected cotton. After calibration, this systematical bias was almost removed. As shown in Fig. 3b, the NDVI of healthy cotton was very similar to the NDVI of infected cotton during the same period. In this period, the mean NDVI of healthy cotton was 0.457, while the mean NDVI of infected cotton was 0.440.

**Figure 4:**
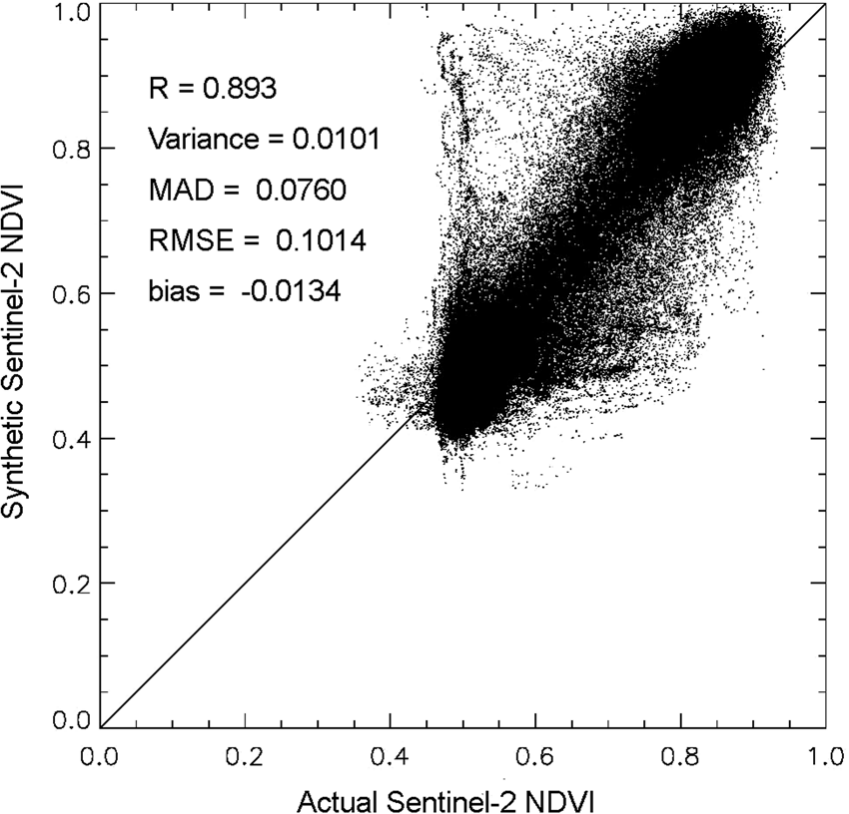
Scatter plot between actual and calibrated synthetic Sentinel-2 NDVI on September 29, 2016.

Figure 4 shows the scatter plot between the actual Sentinel-2 NDVI and the calibrated synthetic Sentinel-2 NDVI along with the accuracy evaluation parameters. Comparing Figs 2 and 4, the *R*-value improved from 0.860 to 0.893, while the *RMSE* reduced from 0.110 to 0.101. Thus, by calibration, the symmetrical bias was removed successfully and the accuracy of synthetic Sentinel-2 NDVI improved.

**Figure 5:**
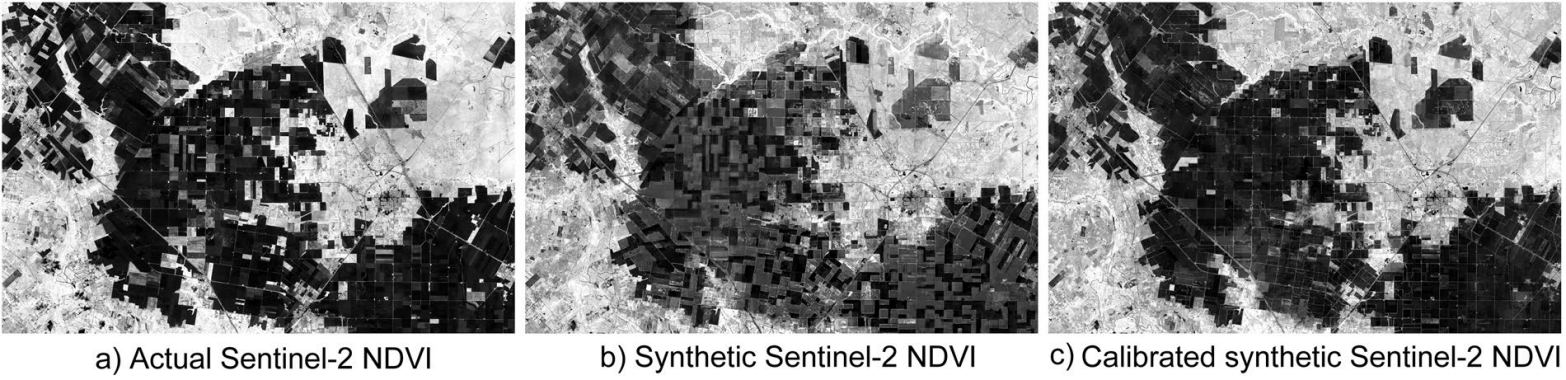
Comparison between (**a**) actual Sentinel-2 NDVI, (**b**) synthetic Sentinel-2 NDVI before calibration and (**c**) calibrated synthetic Sentinel-2 NDVI. (Figure (**a**) was calculated from Sentinal-2 imagery which was downloaded from the web page of the U.S. Geological Survey (USGS) Global Visualization Viewer (http://glovis.usgs.gov/); Figure (**b**,**c**) were calculated from MODIS images and Sentinal-2 imagery which were downloaded from the web page of the U.S. Geological Survey (USGS) Global Visualization Viewer (http://glovis.usgs.gov/); Figure (**a**–**c**) were combined to one Figure using Powerpoint 2013 (Microsoft Inc., Redmond, WA, USA, https://www.microsoft.com)).

Figure 5c shows the calibrated synthetic Sentinel-2 NDVI. Compared with actual Sentinel-2 NDVI (Fig. 5a) and the synthetic Sentinel-2 NDVI before calibration (Fig. 5b), the calibrated synthetic Sentinel-2 NDVI appeared more similar to the actual Sentinel-2 NDVI than the synthetic Sentinel-2 NDVI before calibration. However, there remained some differences between these two NDVI images. This is because the symmetrical bias was estimated using the Sentinel-2 data acquired on February 2, 2016. At this time, all of the cotton and grain crops had not been sown. However, on September 29, 2016, some of the crops were still not harvested as the harvest times of different fields were different.

#### Results of time series monitoring of cotton root rot

**Figure 6:**
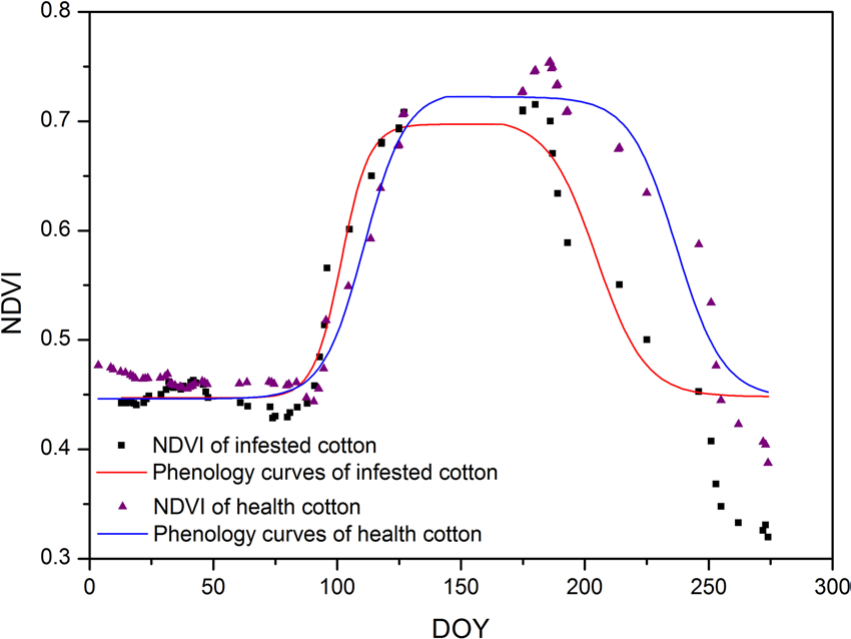
Mean phenology curves of healthy cotton and infected cotton extracted from synthetic Sentinel-2 NDVI time series using a logistic model.

Table 1 shows the fitted results (a, b, c parameters of logistic models and *R*^2^ value) of healthy and infected cotton in the greenness increase and decrease phases, respectively. It can be seen from Table 1 that high accuracies were acquired with *R*^2^ values from 0.791 to 0.969. Figure 6 shows the fitted mean phenology curves of healthy and infected cotton in the measured-fields. Table 2 shows the calculated phenology parameters of healthy and infected cotton. From Fig. 6 and Table 2, the maximum NDVI, the onset day of greenness minimum (OGM), the growing season length (GLS), and integral area value of infected cotton were less than those of healthy cotton. The maximum NDVI of healthy cotton was 0.743, whereas the maximum NDVI was 0.697 for infected cotton. Most of the infected cotton died on average a month before harvest. The GLS of infected cotton was 22 days less than the GLS of healthy cotton. Due to the lower max NDVI, earlier OGM, and shorter GLS, the integral area of infected cotton was 18.54% less than that of healthy cotton, indicating a lower yield for infected cotton^27^.

**Table 1:** Phenology parameters of healthy and infected cotton fitted using a logistic model.

Phase	Healthy cotton			Infected cotton		
	Parameters		*R* ^2^	Parameters		*R* ^2^
Greenness increase phase	*a*	12.742	0.969	*a*	18.120	0.967
	*b*	−0.113		*b*	−0.178	
	*c*	0.296		*c*	0.250	
Greenness decrease phase	*a*	−25.248	0.919	*a*	−22.075	0.791
	*b*	0.107		*b*	0.107	
	*c*	0.275		*c*	0.244

**Table 2:** Parameters extracted from phenology curves of healthy and infected cotton.

Parameters	Healthy cotton	Infected cotton
Max NDVI	0.743	0.697
OGI (day)	101	93
OGM (day)	249	219
GLS (day)	148	126
Integral area	81.27	99.77

## Discussion

**Figure 7:**
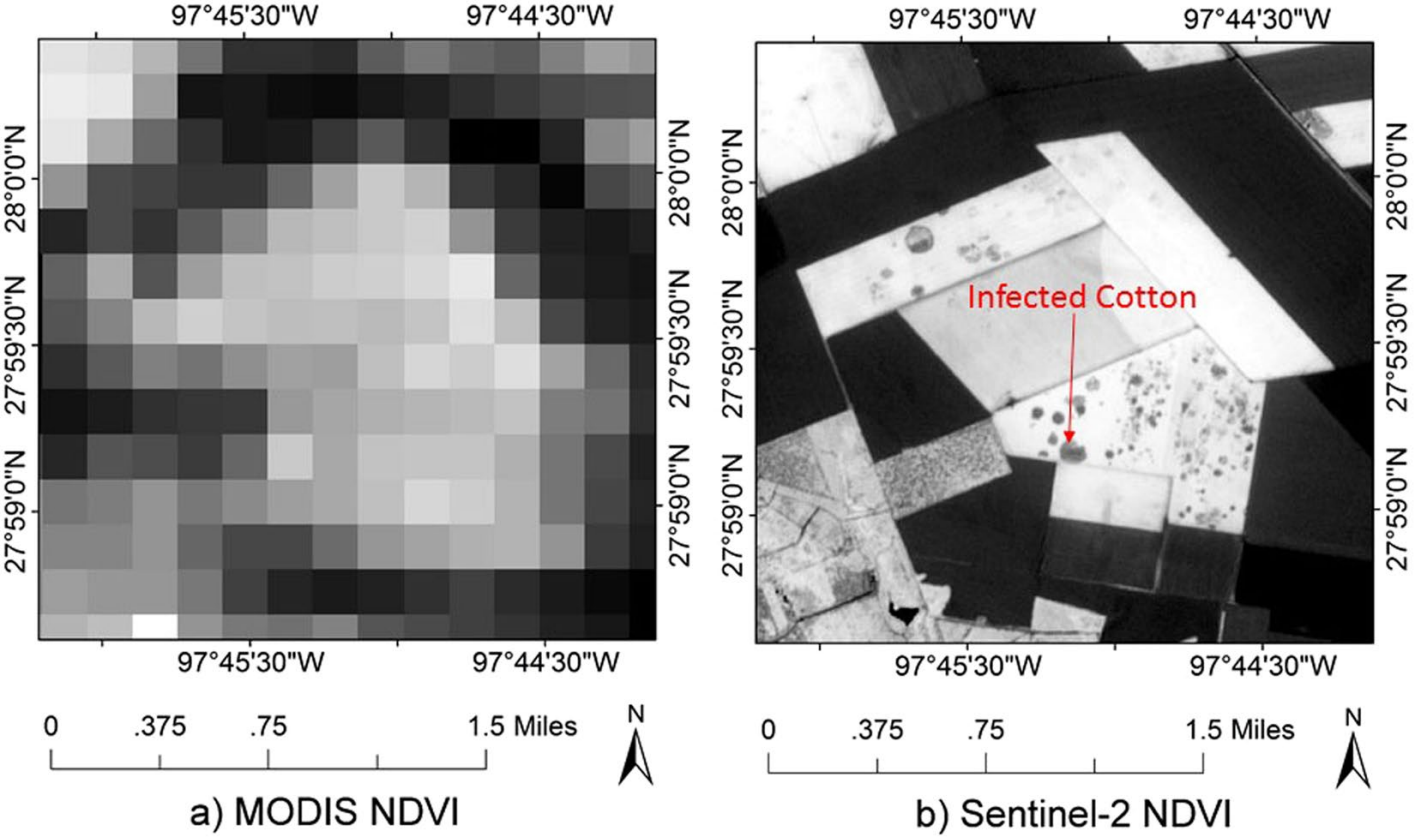
An example of infected cotton in (**a**) MODIS NDVI image and (**b**) Sentinel-2 NDVI image both acquired on June 11, 2016. (Figure (**a**,**b**) were calculated from MODIS images and Sentinal-2 imagery which were downloaded from the web page of the U.S. Geological Survey (USGS) Global Visualization Viewer (http://glovis.usgs.gov/); Figure (**a**,**b**) were combined to one Figure using Powerpoint 2013 (Microsoft Inc., Redmond, WA, USA, https://www.microsoft.com)).

Infected cotton usually has a sporadic distribution within a cotton field (Fig. 7b). The sizes of infected areas vary from a few square meters to several hectares and therefore high spatial resolution imagery is required for monitoring. Thus, due to the lower spatial resolution of MODIS data, infected cotton is always mixed with healthy cotton and smaller infected areas cannot be identified in the 250-m MODIS image (Fig. 7a). Although the temporal resolution of MODIS is high, it alone is not suitable for time series monitoring of cotton root rot.

The spatial resolution of Sentinel-2 NDVI is 10-m. The high spatial resolution makes it suitable to identify cotton root rot (Fig. 7b). However, the revisit time of Sentinel-2 satellite is currently about five days. A longer revisit time and the influence of clouds made it difficult to acquire sufficient cloud-free images for time series monitoring of cotton root rot. For example, there were only five Sentinel-2 images that could be used in this study area (Table 3), whereas the number of MODIS images acquired under clear conditions was 60. The number of Sentinel-2 images was too few to monitor the phenology of cotton, especially for the extraction of such parameters as the onset of greenness increase (OGI), OGM, and GLS. Thus, the synthetic Sentinel-2 images generated by combining MODIS and Sentinel-2 data as described in the results of time series monitoring of cotton root rot section were used for time series monitoring of cotton root rot.

**Table 3:** Available Sentinel-2 images in San Patricio County, Texas from January 1 to September 30, 2016.

Acquired date	Cloud coverage percentage
02/02/2016	0%
04/02/2016	5%
07/11/2016	0%
07/31/2016	50%
09/29/2016	0%

As shown in the results section, the phenology curve of infected cotton showed a significant difference from that of healthy cotton. The max NDVI value, OGM, GSL and the integral area of the phenology curve for infected cotton were reduced by 0.045, 30 days, 22 days, and 18.54%, respectively, compared with those for healthy cotton. Several studies had demonstrated that phenology monitored by these synthetic data had a high accuracy and was very similar to ground observed or MODIS-based phenology^21,23,28^. Thus, cotton root rot will cause a decrease in cotton gross primary production (GPP) that is an important parameter related to crop yield^29^. Since the phenology monitored using synthetic Sentinel-2 time series has 10-m resolution, while the MODIS-based phenology product has 500-m resolution, it can provide more useful information for applications in crop yield estimation^22^. Crop yield estimation using these synthetic data is an important direction for future research.

Although the synthetic Sentinel-2 NDVI time series had a higher spatial resolution than the original MODIS data and a better temporal resolution than the original Sentinel-2 data, there remain some issues that affect the accuracy of this data fusion method or limit its application. First, as different atmospheric correction methods are used for the two sensors, there exist differences in NDVI data between the two sensors. This will reduce the fusion accuracy of ISTDFA. Alternative atmospheric correction methods should be tested in future studies. Second, land cover change reduces the accuracy of ISTDFA^16^. Most spatial and temporal data fusion methods (e.g. STARFM, STDFA and ISTDFA) were not proposed for the situation of land cover change. As a result, these methods showed a low accuracy in areas with land cover change^16,30^. A more serious problem is that it can lead to failure of the sensor difference adjustment method because different land cover types have different sensor difference adjustment parameters. Thus, more reference high spatial resolution images are required to minimize the effect of land cover change during the fusion period. Alternatively, some new spatial and temporal data fusion methods like FSDAF that were specifically designed for land cover change should be used^25^. The third factor is the influence of cloud coverage. Although this method can be used to increase the temporal frequency of high spatial resolution data, cloud-free high temporal resolution images are required. For example, there were no cloud-free MODIS images from May 4 to June 22, 2016, for San Patricio County, TX, USA. One alternative solution is to fill the gaps using radar images which can be acquired during cloudy weather.

## Materials and Methods

### Study area

**Figure 8:**
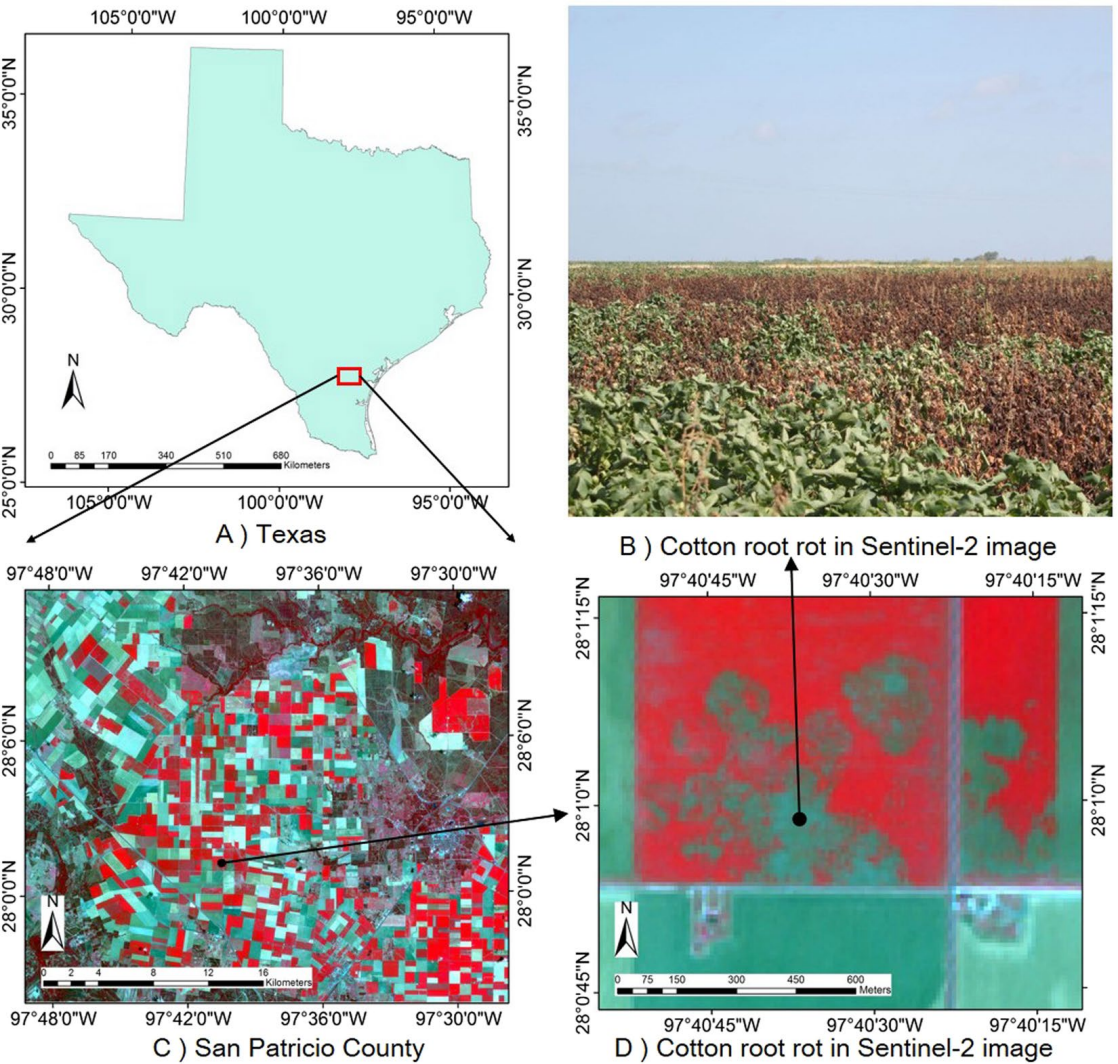
Location of the study area with Sentinal-2 color-infrared image (NIR, Red and Green bands) acquired on July 11, 2016 and provided by the U.S. Geological Survey (USGS). (Figure (**A**,**C**,**D**) were generated using ARCGIS 10.2 (Esri Inc., NY, USA, http://www.esri.com/); Figure (**B**) was acquired with Nikon D810 digital CMOS cameras (Nikon Inc., Melville, NY, USA, http://www.nikonusa.com); Figure (**A**–**D**) were combined to one Figure using Powerpoint 2013 (Microsoft Inc., Redmond, WA, USA, https://www.microsoft.com)).

A 45 km × 29 km rectangular area in San Patricio County, TX, USA, was selected as the study area (Fig. 8). The latitude and longitude of this area range from 27°55′39.92″N to 28°11′38.86″N and 97°51′19.60″W to 97°23′39.43″W, respectively. The topography of the study area is generally flat. The main land cover types are crops (cotton, corn, and grain sorghum), grass, trees, water, and residential areas. The area has a history of serious cotton root rot.

### Data and preprocess

#### Sentinel-2

Sentinel-2 consists of two identical satellites, Sentinel-2A and Sentinel-2B, developed by the European Commission’s Copernicus Programme. Similar to the Chinese Huanjing (HJ) satellite constellation, which was launched on September 6, 2008, Sentinel-2 has two wide-swath high-resolution multispectral imagers onboard. However, the multispectral imagers onboard the Sentinel-2 satellites have 13 spectral bands, whereas the HJ imagers only have four spectral bands. Sentinel-2A was launched on June 23, 2015 and Sentinel-2B was launched on March 7, 2017. The parameters of Sentinel-2 and HJ imagers are shown in Table 4.

**Table 4:** Parameters of Sentinel-2 and HJ satellite imagers.

Sentinel-2					HJ				
Band	Center Wavelength (µm)	Resolution (m)	Width (km)	Revisit Time (Day)	Band	Center Wavelength (µm)	Resolution (m)	Width (km)	Revisit Time (Day)
1	0.443	60	290	5				360	2
2	0.490	10			1	0.475	30		
3	0.560	10			2	0.560	30		
4	0.665	10			3	0.660	30		
5	0.705	20							
6	0.740	20							
7	0.783	20							
8	0.842	10			4	0.830	30		
8A	0.865	20							
9	0.945	60							
10	1.375	60							
11	1.610	20							
12	2.190	20							

Three Sentinel-2A images acquired on February 2, July 11, and September 29, 2016, under clear sky conditions, were used in this study. These images with L1C processing were downloaded from the web page of the U.S. Geological Survey (USGS) Global Visualization Viewer (http://glovis.usgs.gov/). The images were cropped to the study area and converted to ENVI format using the European Space Agency’s Sentinel-2 Toolbox. Then, the images were atmospherically corrected using the Quick Atmospheric Correction tools in ENVI 5.3. Finally, the NDVIs for each date were calculated. The Sentinel-2 image acquired on July 11, 2016 was used as the reference image in the fusion. The Sentinel-2 image acquired on September 29, 2016 was used for the validation of the synthetic Sentinel-2 image. The Sentinel-2 image acquired on February 2, 2016 was used for the calibration of the synthetic Sentinel-2 time series.

#### MODIS

Sixty 250-m MODIS daily surface reflectance datasets (MOD09GQ) acquired under clear sky conditions from January 1 to September 30, 2016, were used in this study. These MODIS datasets were downloaded from the web page of the USGS Global Visualization Viewer (http://glovis.usgs.gov/) in a higher-order gridded level-2 (L2G) and Version-6. The MOD09GQ product was corrected for atmospheric gases and aerosols. The horizontal and vertical tile numbers of MODIS in San Patricio County are 9 and 6, respectively. All of these MODIS data were cropped to the study area and projected to the UTM-WGS84 reference system using MODIS Reprojection Tool (MRT) software. Then the time series MODIS NDVIs were calculated.

#### Airborne data

Airborne multispectral images with red, green, blue and NIR bands were acquired on July 20, 2016 to identify the infected areas of cotton root rot using the visual interpretation method along with the field-measured land cover type data. The airborne images were collected at an altitude of approximately 3000 m above ground level with two Nikon D810 digital CMOS cameras onboard a Cessna 206 aircraft^31^. One camera was used to acquire the RGB image, and the other to acquire the NIR image. Then these images with a pixel array of 7360 × 4912 were mosaicked to a 0.8-m orthomosaic image using Pix4Dmapper software (Pix4D, Lausanne, Switzerland) with 23 field measured ground control points (GCPs)^32^.

#### Field data

Two types of field data were collected by a field survey with a GPS with an accuracy of 1–8 cm, on August 24, 2016. The first type of field data was the 23 field-measured GCPs that were relatively evenly distributed in this study area and were easily identifiable in the airborne imagery. The second type of field data were land cover types. Land cover types, in particular the infected areas of cotton, were recorded in the airborne imagery. A total of 48 fields or areas covering the main land cover types (healthy cotton, infected cotton, and corn/grain sorghum) were ground verified.

### Generation of synthetic Sentinel-2 NDVI time series using ISDTFA

#### Introduction of ISDFA

According to unmixing theory, the reflectance or NDVI of a coarse-resolution spatial pixel is a linear combination of the responses of each land-cover class contributing to the mixture, which can be described as follows^33^:

1 $$R(i,t)=\sum _{c=0}^{m}{f}_{c}(i,c)\times \overline{r}(c,t)+\xi (i,t)$$

$${\rm{Constrained}}:\,{\sum }_{c=0}^{m}{f}_{c}(i,c)=1;\,{\rm{and}}\,1\ge {{f}}_{{c}}(i,\,c)\ge 0\,{\rm{for}}\,{\rm{all}}$$

where *R(i, t)* is the reflectance or NDVI of a coarse-resolution spatial pixel *i*; $\overline{r}(c,t)$ is the mean value of land cover class *c* at time *t*; *f*_*c*_*(i*, *c)* is the fractional cover of class *c* in coarse pixel *i*; and $\xi (i,t)$ is the residual error term. Using ordinary least squares techniques, the time series mean value (reflectance or NDVI) of the finer resolution data of each land cover type can be estimated from the time series coarser resolution data^34^. Based on the assumption that the temporal variation properties of each pixel belonging to the same land cover type is similar to the mean value of the temporal variation properties, the STDFA model combines coarser and finer resolution data to generate temporal finer resolution data as follows^15^:

2 $$r(k,c,{t}_{i})=\overline{r}(c,{t}_{i})-\overline{r}(c,{t}_{0})+r(k,c,{t}_{0})$$

where $\overline{r}(c,{t}_{0})$ and $\overline{r}(c,{t}_{i})$ are the mean values of land cover class *c* at time *t*_0_ and *t*_*i*_, respectively, which can be estimated from the time series coarser resolution data using ordinary least squares techniques and the fractional cover of each land cover type; *r(k*, *c*, *t*_0_) and *r(k*, *c*, *t*_*i*_) are the values of the *k*th pixel in class *c* at time *t*_0_ and *t*_*i*_, respectively. Usually, *r(k*, *c*, *t*_0_) is obtained from the finer resolution data at time *t*_0_ and *r(k*, *c*, *t*_*i*_) is the value to be estimated. To address the problems of sensor differences and spatial variability, Wu *et al*. improved STDFA (ISTDFA), which is described as follows^16^:

3 $$r(k,c,{t}_{i})=a\times (s(i,j,{t}_{i})\times \overline{r}(c,s,{t}_{i})-s(i,j,{t}_{0})\times \overline{r}(c,s,{t}_{0}))+r(k,c,{t}_{0})$$

where $\overline{r}(c,s,{t}_{0})$ and $\overline{r}(c,s,{t}_{i})$ are the mean values of land cover class *c* at time *t*_0_ and *t*_*i*_, respectively, in subset *s* which is estimated using a weighted linear mixed model with information from neighboring pixels to address the problem of surface reflectance spatial variability; *a* is the slope of the linear method between the mean values of the finer and coarser resolution data built using a linear-regression method to remove the influence of differences in sensor systems; and *s(i*, *j*, *t*_0_) and *s(i*, *j*, *t*_*i*_) are the spatial-variability adjustment factors of coarser pixel *j* for coarser MODIS pixel *i* of land-cover class *c* at time *t*_0_ and *t*_*i*_, respectively, in subset *s*.

#### Application of ISDFA

Numerous studies have proved that spatial and temporal data fusion methods such as STDFA, STARFM, and ESTARFM, can be used to fuse NDVI data^13,15,35^. Moreover, the vegetation index fusion strategy is more suitable than the reflectance fusion strategy when applying these methods to NDVI data fusion^35,36^. Thus, ISTDFA was applied and validated for the fusion of MODIS and Sentinel-2 NDVI data. There are three inputs in ISTDFA: land cover type, a finer resolution data used as the reference image, and the time series coarser resolution image. The Sentinal-2 NDVI image acquired on July 11, 2016 was used as the reference image. A land cover type classification map was created from the multispectral Sentinal-2 image acquired on July 11, 2016 using the maximum likelihood method, with field-measured land cover type data as regions of interest (ROIs). Sixty 250-m MODIS NDVI images, acquired from January 1 to September 30, 2016, were used as the time series coarser resolution data. By inputting these data, sixty 10-m synthetic Sentinal-2 NDVI images were generated by the ISTDFA method.

#### Validation of generated synthetic Sentinel-2 NDVIs

The object of spatial and temporal data fusion is to generate synthetic time series finer spatial resolution data. Thus, the similarity between synthetic and actual finer spatial resolution data is used to evaluate the accuracy of data fusion methods. Both qualitative and quantitative methods can be used to evaluate the performance of data fusion methods. Visual interpretation is the most widely used qualitative method. Obviously, the smaller the difference between the synthetic and actual data, the more accurate the method is. Parameters such as *R*, variance, *MAD*, *bias*, and *RMSE* are the most widely used parameters to quantitatively evaluate the performance of data fusion methods. Higher *R-*values and lower values of variance, *MAD*, *bias* and *RMSE* indicate higher accuracy of the methods. The scatter plot is another widely used method to quantitatively evaluate these methods. Higher *R*-values in the scatter plot indicate a greater similarity in the two datasets and a higher accuracy of the data fusion methods. All these methods are widely used in the evaluation of spatial and temporal data fusion methods such as STDFA, ISTDFA, STARFM, and ESTARFM^12,13,15,16^. Thus, they were also used to evaluate the performance of ISTDFA in the fusion of 250-m MODIS and 10-m Sentinal-2 NDVI data. Actual 10-m Sentinal-2 NDVI data acquired on September 29, 2016, was used to evaluate the accuracy of ISTDFA using the above methods.

### Time series monitoring of cotton root rot

#### Calibration of synthetic Sentinel-2 NDVI time series

When ISTDFA was applied to combine the time series MODIS and Sentinal-2 NDVI data, time series synthetic 10-m Sentinal-2 NDVI data were generated. Thus, time series NDVI curves of the different land cover types were extracted and used for time series monitoring. However, for various reasons, the values of the same land cover types in a synthetic Sentinel-2 image may be different. For example, the cotton and corn fields in February were bare as the fields were not planted in the study area. The NDVI values of bare land for cotton and corn areas should be similar. However, the NDVI values may be different in the synthetic Sentinal-2 NDVI time series for the following reasons:

1. Difference in atmospheric correction methods. The MODIS data were atmospherically corrected by the 6 S radiative transfer code, whereas the Sentinel-2 data were atmospherically corrected by Quick Atmospheric Correction tools in ENVI 5.3. As different atmospheric correction methods were used, the NDVI values of the same land were different. For example, the NDVI value of bare land in location (28°04′03.39″N, 97°40′57.29″W) is 0.229 in the MODIS data, whereas it is 0.455 in the Sentinel-2 data. Thus, the sensor difference was magnified by different atmospheric correction methods.
2. Influence of land cover change. From January 1 to September 30, 2016, the cover types of the crop land changed in the following order: bare soil (before sowing), crops (cotton, corn and grain sorghum), and bare soil with or without residuals (after harvest). However, the crops were set to be either cotton or grain (corn and grain sorghum) only when applying ISTDFA. Moreover, the sensor difference adjustment parameter *a* for the crop types was used for all datasets from January 1 to September 30, 2016, which was different for different land cover types.
3. Influence of the solution of Equation (1). Because the bare soil was divided into three land cover types (healthy cotton, infected cotton, and grain), the disaggregated mean values of these three land cover types before sowing and after harvest may be different, leading to a difference in the synthetic Sentinel-2 data. Thus, it was necessary to calibrate the NDVI of the same land cover types to the same value.

Although the factors for the differences in the same coarser land cover types are complex and it is difficult to show which factor has the greatest influence, the biases caused by these factors are systematic, enabling them to be removed easily. Therefore, a relatively simple calibration method was proposed and used to calibrate the synthetic Sentinel-2 NDVI time series. The method was carried out by three steps. First, an actual and a synthetic Sentinel-2 NDVI image, both from February 2, 2016, were used to estimate the bias image, which was equal to the difference in the images. Then, each image in the time series was calibrated by subtracting this difference image from every synthetic Sentinel-2 NDVI image. Finally, the method described in the validation of generated synthetic Sentinel-2 NDVIs section was used to evaluate the calibrated results again.

#### Phenology monitoring of cotton root rot

Cotton root rot occurs continuously from June to September when the temperature is high in the study area. When cotton is infected, its leaves will become yellow and then brown. This will lead to a reduction of NDVI in the images. Thus, time series monitoring of NDVI is a good method to detect cotton root rot. It can be conducted in three steps, the first of which is data smoothing. To remove the noise, a Savitzky–Golay filter was applied to smooth the synthetic Sentinel-2 NDVI time series. Then, the phenology of infected and healthy cotton was detected using a widely used logistic model, described as follows^37,38^:

4 $$NDVI(t)=\frac{c}{1+{e}^{a+b\times t}}+NDV{I}_{b}$$

5 $$NDVI(t)=\frac{{c}_{2}+dt}{1+{e}^{{a}_{2}+{b}_{2}t}}+NDV{I}_{b}$$

where *NDVI*_*b*_ is the background NDVI; *t* is the time of day of the year (DOY). Equation (4) is used for favorable growth conditions. Equation (5) is used for vegetation under stress. The mean value before March 14, 2016 when the crops had not been sown was set to the value of *NDVI*_*b*_. Then, this model was applied to fit the NDVI time series in the greenness increase and decrease phases, respectively. Finally, the NDVI time series phenology curves of infected and healthy cotton were compared by several parameters. The first parameter is the max NDVI value in the phenology curve, which represents the maximum greenness the cotton can reach. Usually, the higher the maximum NDVI, the healthier the cotton plants are. The second parameter is OGM. The infected cotton will have a lower maximum NDVI value and an early OGM. The third parameter is GLS, which is defined as the days between OGI and OGM. The OGI and OGM are the transition dates, which can be identified using the maximum values in the rate of change of curvature^38,39^. The infected cotton will die earlier and have a shorter GLS. The fourth parameter is the integral area during the growing season, which begins at OGI and ends at OGM. The integral area of the infected cotton will be less than the integral area of healthy cotton due to the lower max NDVI, earlier OGM, and shorter GLS.
